# Comparison between day surgery and non-day surgery in the procedure for prolapse and hemorrhoids (grades III–IV) with MRI-assisted diagnosis: a retrospective cohort study

**DOI:** 10.3389/fmed.2025.1653122

**Published:** 2025-08-15

**Authors:** Shaohua Zhang, Yanbin Zhao, Yifan Wei, Guodong Jing, Youyu Luo, Shaoting Zhang, Liqiang Hao, Yonggang Hong

**Affiliations:** ^1^Department of Colorectal Surgery, Changhai Hospital, Shanghai, China; ^2^Department of Radiology, Changhai Hospital, Shanghai, China; ^3^Department of Radiology, Shandong Cancer Hospital and Institute, Jinan, China

**Keywords:** mixed hemorrhoids, PPH, day surgery, ambulatory surgery, feasibility

## Abstract

**Background:**

To evaluate the clinical value of day surgery with MRI-assisted diagnosis for the procedure for prolapse and hemorrhoids (PPH) through a retrospective cohort study. MRI was included in the preoperative protocol for surgical planning.

**Methods:**

A total of 107 patients who underwent day surgery PPH with preoperative perianal magnetic resonance imaging (MRI) for mixed hemorrhoids from October 2021 to July 2023 and 234 patients who underwent non-day surgery from April 2008 to April 2023 were included in this retrospective analysis. Outcomes of the two groups were compared, including intraoperative blood loss, post-discharge pain scale, time to resume normal activities, postoperative complications, healing of the anastomosis and wounds, discharge satisfaction rate, and short-term recurrence rate.

**Results:**

The day surgery group experienced significantly less intraoperative blood loss compared to the non-day surgery group [10 (5–20) ml vs. 20 (20–50) ml, *p* < 0.01]. The post-discharge pain scale was slightly higher in the day surgery group (*p* = 0.041). The discharge satisfaction rate was higher in the day surgery group (97.2% vs. 90.6%, *p* = 0.030). Patients in the day surgery group resumed normal activities earlier than those in the non-day surgery group [20 (14–30) days vs. 30 (14–30) days, *p* = 0.003]. The rate of postoperative residual tissue prolapse was lower in the day surgery group (0.9% vs. 6.0%, *p* = 0.035). No significant differences were observed between the groups in terms of anastomosis and wound healing, short-term recurrence rates, or other postoperative complications (all *p* > 0.05).

**Conclusion:**

Day surgery with MRI-assisted diagnosis for mixed hemorrhoids is effective, feasible, and associated with shorter hospitalization times, higher patient satisfaction, faster recovery, improved resource efficiency, and enhanced bed turnover. It is a promising model worthy of clinical adoption.

## Introduction

Mixed hemorrhoids are a common benign anal disease that can occur at any age, with incidence increasing with age. Risk factors include habitual constipation, prolonged sitting or standing, alcohol consumption, stimulating food, and pregnancy (1). The primary clinical symptoms of mixed hemorrhoids include bleeding, hemorrhoid prolapse, heavy pain, distending pain, perianal itching, and abnormal perianal secretion. Bleeding is a hallmark symptom, especially in the early stages, often appearing as blood on stool or toilet paper, which can progress to dripping blood. In severe cases, patients may experience spraying hemorrhage, potentially leading to anemia.

Hemorrhoid prolapse is common in advanced cases. In mild cases, hemorrhoids prolapse during defecation but retract spontaneously. If uncontrolled, manual reduction is necessary. Severe cases involve prolapse triggered by increased abdominal pressure (e.g., coughing, sneezing, exertion) that is difficult to reduce. Surgery becomes necessary when conservative treatments fail.

The procedure for prolapse and hemorrhoids (PPH) is a common surgical approach for treating grade III and IV internal hemorrhoids and mixed hemorrhoids. PPH offers advantages over traditional surgery in managing outlet obstructive constipation caused by internal rectal prolapse with circumferential hemorrhoids (2, 3). During this minimally invasive procedure, the surgeon excises the mucous membrane and hemorrhoidal artery 2–3 cm above the hemorrhoid using a stapler. The stapler also lifts the rectal mucous membrane and hemorrhoidal tissue for anastomosis, resolving prolapse by preventing the hemorrhoidal core from protruding.

Procedure for prolapse and hemorrhoids causes less surgical damage, making it suitable for elderly patients and those with rectal mucosal prolapse. It facilitates quicker recovery and reduces recurrence for patients with a history of traditional surgery (4). However, PPH is not universally applicable; physicians evaluate patient conditions to determine its suitability (5).

Day surgery is an emerging treatment model in which patients complete hospitalization, surgery, and discharge within 24 h. This model is increasingly adopted in modern medical practice (6). Although day surgery for PPH shows high feasibility, further evidence is needed to validate its effectiveness (7). This study reports on 107 patients who underwent day surgery PPH and critically analyzes its clinical outcomes and feasibility.

## Materials and methods

### Study population and design

This study included 107 patients with mixed hemorrhoids who underwent day surgery PPH at Changhai Hospital between October 2021 and July 2023, along with 234 patients who underwent non-day surgery PPH between April 2008 and April 2023. The timeline discrepancy between the two groups reflects the functional transformation of China’s tertiary hospital with practical significance, over 95% of our department’s inpatient beds were prioritized for malignant tumor surgeries. Follow-up assessments were conducted through telephone interviews (1 year postoperatively) and outpatient visits (1 week and 1 month postoperatively). Patients were evaluated for post-discharge pain scale (VRS), time to resume normal activities, postoperative complications, discharge satisfaction rate, and short-term recurrence rate.

The study adhered to the Declaration of Helsinki and received approval from the hospital’s ethics committee. Written informed consent was obtained from all participants.

### Inclusion criteria

1. All patients were adults presenting with prolapse symptoms of internal, external, or mixed hemorrhoids (grade III and IV);
2. All patients underwent preoperative perianal MRI.
3. All patients had full capacity for civil acts and could accurately follow medical advice.
4. No uncontrolled hypertension, diabetes, or other severe systemic diseases.

### Exclusion criteria

1. Patients with perianal rectal abscess, perineal gangrene, anal rectal stenosis, or inflammatory bowel disease;
2. Patients who had recently (within 3 months) undergone sclerosant injection or anorectal surgery;
3. Patients with immunodeficiency, coagulopathy, or those requiring ongoing anticoagulant therapy;
4. Pregnant women, children, and patients with refractory constipation, pelvic tumors, portal hypertension, Buerger’s syndrome, or those unable to tolerate surgery.

## Surgery

### Preoperative preparations

1. Preoperative examinations included blood, urine, routine fecal occult blood tests, coagulation profiles, colonoscopy, abdominal ultrasound, and perianal MRI.
2. All patients received health education, were informed about surgical risks, and signed consent forms.
3. Preoperative bowel and bladder emptying were conducted. Routine oral laxatives were not administered; patients with constipation used enemas for bowel cleansing.
4. Patients refrained from food and water after 22:00 the night before surgery.

### Perianal MRI

**FIGURE 1:**
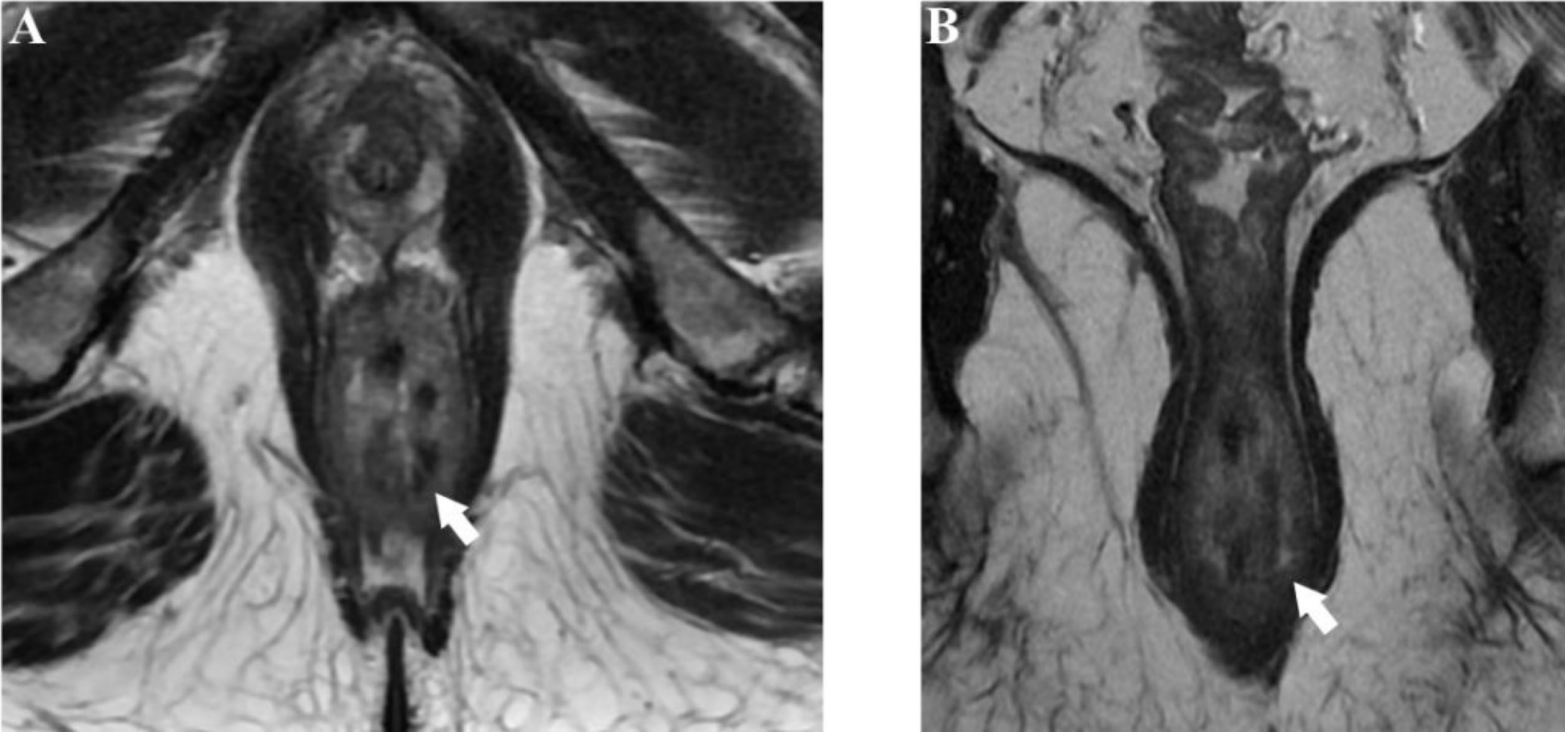
Preoperative perianal magnetic resonance imaging (MRI) of a 43 years-old man. **(A)** Axial T2-weighted and **(B)** coronal T2-weighted image images without fat suppression show a mixed hemorrhoid of anal canal (arrow).

Preoperative perianal MRI was performed via an abdominal phased-array coil. The main imaging protocol included sagittal T2-weighted imaging (T2WI), axial high-resolution T2WI, coronal high-resolution T2WI, and gadolinium contrast-enhanced axial, sagittal, and coronal T1-weighted imaging. The diagnosis of hemorrhoids was determined by two radiologists with more than 10 years’ diagnostic experience (GD. J. and ST. Z.). Any discrepancies between the two radiologists were resolved by discussion. The MRI findings were used to assess surgical complexity, detect coexisting anal pathologies, and assist in individualized planning. However, MRI findings were not used as discharge criteria nor as a determinant for postoperative recovery. Example images shown in Figure 1.

### Surgical procedures

Studies have shown that stapled hemorrhoidopexy can be safely performed as a day surgery procedure (8). Based on patients’ conditions and MRI performance, surgeons may choose other surgical methods as needed. The most common approach is PPH combined with Milligan-Morgan hemorrhoidectomy (9), with occasional cases involving anal fistulectomy or anorectal polypectomy (10). All surgical trauma is balanced and comparable among the procedures.

All surgeries were performed under lumbar anesthesia combined with nerve block. Early complications of spinal anesthesia, including hypotension, nausea and vomiting, urinary retention, post-dural puncture headache, and prolonged motor block, were carefully monitored and proactively managed. Anesthesia and nursing teams adhered to strict perioperative protocols to minimize these risks in the day surgery setting. Day surgeries were conducted by one experienced surgeon group (led by a deputy chief physician, with an annual volume of approximately 280 benign anal disease cases and 150 colorectal cancer cases). Non-day surgeries were conducted by the same surgeon group.

The basic PPH day surgery procedure is described below:

- The patient was placed in the lithotomy position. Following anesthesia, routine surgical disinfection and sterile draping were performed.
- The anus and rectum were disinfected, and the anus was dilated with a finger or a circular anal dilator.
- Using three non-traumatic forceps, the anal margin skin at the largest hemorrhoids was clamped to induce slight prolapse of the internal hemorrhoids (avoiding bleeding).
- The dilator and its inner core were inserted into the anus, and the inner core was removed. The dilator was fixed with sutures at 1, 4, 7, and 11 o’clock positions.
- A suture anoscope was inserted into the anus, and a circular suture was made 3–4.5 cm above the dentate line in the rectal submucosa to create a purse-string.
- The PPH stapler was opened to its maximum extent, and its head was inserted above the purse-string suture through the dilator. The suture was gradually tightened and knotted. The sutures were pulled through the stapler’s side holes using the needle holder, drawing the ligated mucosa and submucosa into the stapler’s center rod.
- The stapler was tightened and fired, simultaneously cutting and suturing the mucosa and submucosa above the internal hemorrhoids. The stapler remained closed for 20 s before being fully opened and removed.

The anastomosis site was inspected for bleeding. Any bleeding (active arterial or oozing) was treated with an eight-shaped suture to ensure complete hemostasis. After removing the dilator, Vaseline gauze was inserted. A routine eight-shaped suture was recommended around the anastomosis site, completing the surgery. Surgeons determined whether to combine the procedure with Milligan-Morgan hemorrhoidectomy.

An illustrative case involves a patient with grade IV mixed hemorrhoids successfully treated with day surgery PPH combined with Milligan-Morgan hemorrhoidectomy.

### Postoperative treatment

Patients in the day surgery group to were transferred the daily care ward immediately after surgery, while those in the non-day surgery group were sent to the colorectal and anal surgery department, which had doctors on duty and provided twice-daily rounds by surgeons;

After the operation, all patients remained in a horizontal position without a pillow for 6 h and fasted for 24 h;

Routine intravenous parecoxib and propacetamol were administered for anti-inflammatory and analgesic purposes;

Patients were monitored postoperatively for common complications of lumbar anesthesia. Hypotension was managed with fluid resuscitation and vasopressors if necessary; antiemetics were administered to control nausea and vomiting. Patients were assessed for urinary retention, and catheterization was available when needed. Post-dural puncture headache was evaluated prior to discharge, with extended observation provided if symptoms were suspected. Motor block resolution was a prerequisite for ambulation assessment;

In the day surgery group, dressings and anal tubes were removed the next day. Patients were discharged if there was no significant bleeding, urination was normal, and they felt well. In the non-day surgery group, the chief physician determined discharge time based on the patient’s condition.

### Discharge guidelines

Health education materials and a paper discharge summary were provided to all patients. Discharge criteria included the absence of spinal anesthesia-related complications: patients were required to be fully alert, able to ambulate independently, void urine normally, and report no significant headache, nausea, or motor weakness;

A 7 days supply of celecoxib (11) and diosmin (12) was prescribed, with instructions to take the medication as directed, maintain a light diet, and ensure bowel regularity (1–2 defecations per day);

Patients were advised to take sitz baths in warm water or a 1:5000 potassium permanganate solution for 20–30 min after defecation, at least twice daily, and to remain seated during the bath;

All patients were required to attend follow-up visits on days 7, 14, and 30 postoperatively, with additional visits scheduled in case of discomfort.

### Statistical analysis

SPSS 26.0 statistical software was used for data analysis. Enumeration data were described as [*n* (%)]. Measurement data were first tested for normality using the Shapiro-Wilk test, with non-normally distributed data represented as [M (P25, P75)]. Differences between the non-day surgery and day surgery groups for mixed hemorrhoids were analyzed using the Chi-square test or Mann-Whitney U test. The significance level was set at α = 0.05, with *P* < 0.05 considered statistically significant.

## Results

### Patient features

**FIGURE 2:**
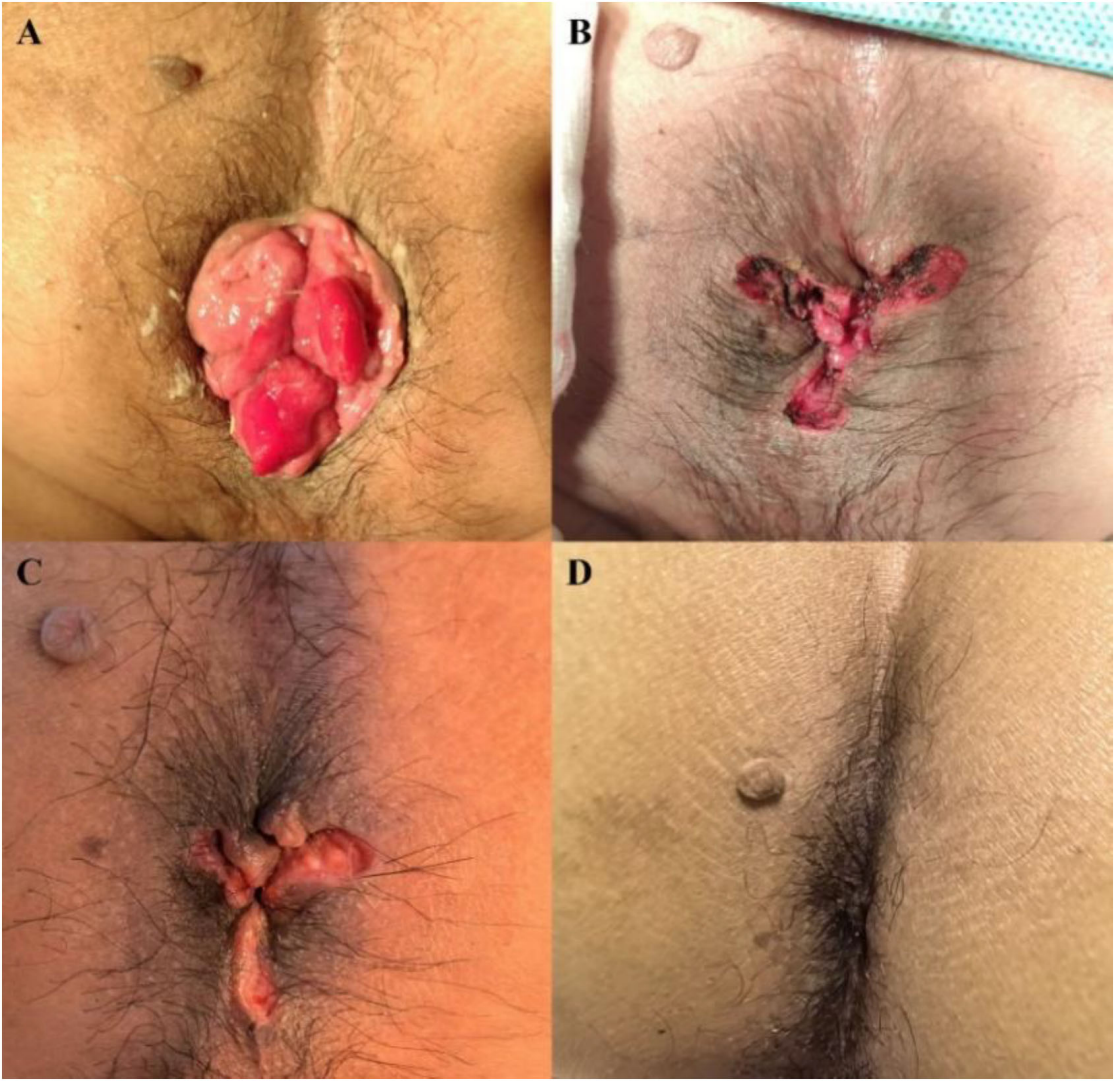
**(A)** Condition before surgery (grade IV mixed hemorrhoids); **(B)** Postoperative condition (wound appears flat); **(C)** 1 week after surgery (wound recovery is progressing well); **(D)** 1 month after surgery (healed wound with no evidence of prolapsed tissue).

**FIGURE 3:**
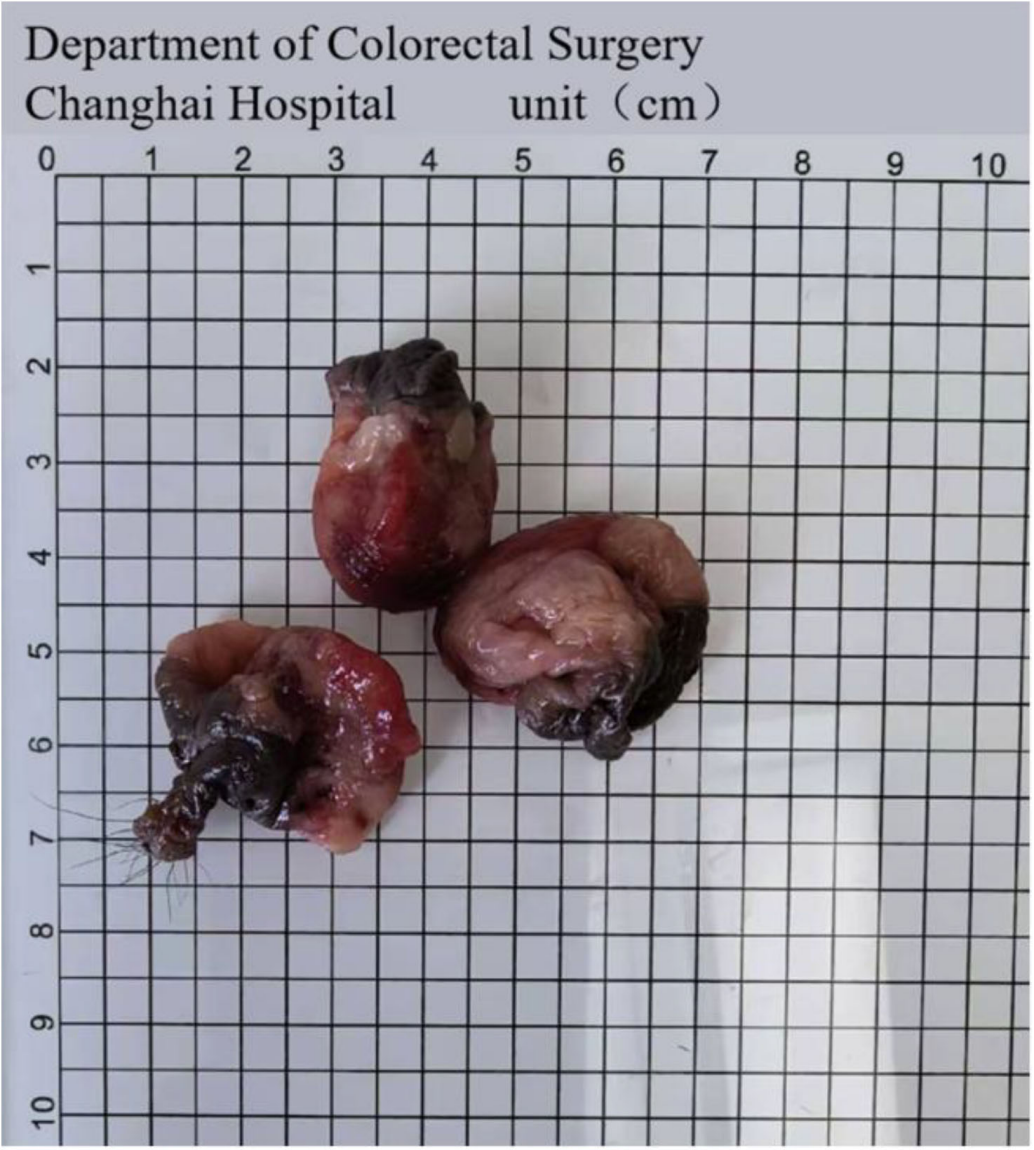
Resected tissues.

The comparison between the non-day surgery and day surgery groups showed that the hospital stay length was also shorter for the day surgery group [2 (1, 2) days vs. 6 (5, 8) days, *p* < 0.001]. In the day surgery group, patients who stayed up to 2 days were included, as some required brief observation due to late surgical timing or minor postoperative concerns. There was no statistical difference in gender composition, age or BMI between the groups (*p* > 0.05). Detailed patient features are presented in Table 1. Example case shown in Figures 2, 3.

**TABLE 1:** Variance analysis of general information [*n* (%), M (*P*_25_, *P*_75_)].

Item	Value	Non-day surgery group	Day surgery group	Stats	*P*
Sex	Male	146 (62.4)	57 (53.3)	2.536	0.111
	Female	88 (37.6)	50 (46.7)	–	–
Age		45 (39, 58)	43 (32, 48)	−1.175	0.061
BMI (Kg/m^2^)		23.4 (21.25, 24.6)	23.44 (21.19, 25.25)	−0.793	0.428
The length of hospital stays		6 (5, 8)	2 (1, 2)	−14.963	< 0.001

### Analysis of surgical indicators

Variance analysis of surgical indicators revealed significant differences in the composition of surgical approaches between the non-day surgery and day surgery groups (*P* < 0.001). Due to the extensive tissue damage and severe pain associated with Milligan-Morgan hemorrhoidectomy and the suboptimal outcomes of PPH alone, most patients required treatment using a combination of PPH and Milligan-Morgan hemorrhoidectomy (13).

The proportion of patients undergoing only PPH was lower in the day surgery group compared to the non-day surgery group (34.6% vs. 64.1%, *P* < 0.001). This difference in procedure composition may represent a confounding factor in outcome comparisons. Intraoperative blood loss was also significantly lower in the day surgery group [10 (5, 20) ml vs. 20 (20, 50) ml, *P* < 0.001].

There were no statistically significant differences in wound healing or postoperative bleeding between the two groups (*P* > 0.05). The detailed variance analysis of surgical indicators is provided in Table 2.

**TABLE 2:** Variance analysis of surgical indicators between day surgery group and non-day surgery group [*n* (%), M (*P*_25_, *P*_75_)].

Item	Value	Non-day surgery group	Day surgery group	Stats	*P*
Surgical method	PPH only	150 (64.1)	37 (34.6)	25.841	< 0.001
	PPH + others	84 (35.9)	70 (65.4)	–	–
Healing situation*	Bad	3 (1.3)	0 (0)	2.017	0.365
	Average	2 (0.9)	2 (1.9)	–	–
	Good	229 (97.9)	105 (98.1)	–	–
Postoperative bleeding	No	224 (95.7)	100 (93.5)	4.917	0.086
	Minor bleeding	6 (2.6)	7 (6.5)	–	–
	Major bleeding (Requiring intervention)	4 (1.7)	0 (0)	–	–
Intraoperative blood loss (ml)		20 (20,50)	10 (5,20)	−7.564	< 0.001

*Healing was assessed during outpatient follow-up by the chief surgeon and classified as: Good, complete closure, no infection, minimal inflammation; Average, partial closure, mild inflammation, delayed but progressing healing; Poor, dehiscence, infection, or complications requiring intervention.

### Analysis of prognostic factors for the patients

Variance analysis of post-discharge recovery indicators revealed significant differences between the non-day surgery and day surgery groups. The return visit rate was significantly higher in the day surgery group compared to the non-day surgery group (74.8% vs. 18.8%, *P* < 0.001). Both groups demonstrated a zero short-term recurrence rate.

The post-discharge pain score (VRS) was higher in the day surgery group than in the non-day surgery group (*P* = 0.041). Patient satisfaction rates were also higher in the day surgery group compared to the non-day surgery group (97.2% vs. 90.6%, *P* = 0.030). Additionally, the recovery time was shorter in the day surgery group [20 (14, 30) days vs. 30 (14, 30) days, *P* = 0.003].

The detailed variance analysis of post-discharge recovery indicators is presented in Table 3.

**TABLE 3:** Variance analysis of post discharge recovery indicators between day surgery group and non- day surgery group [*n* (%), M (*P*_25_, *P*_75_)].

Item	Value	Non-day surgery group	Day surgery group	Stats	*P*
Return visit	No	190 (81.2)	27 (25.2)	99.374	< 0.001
	Yes	44 (18.8)	80 (74.8)	–	–
Post discharge pain scale (verbal rating scale, VRS)*	No pain	53 (22.6)	16 (15.0)	8.262	0.041
	Mild pain	104 (44.4)	40 (37.4)	–	–
	Moderate pain	53 (22.6)	39 (36.4)	–	–
	Severe pain	24 (10.3)	12 (11.2)	–	–
Discharge satisfactory	No	22 (9.4)	3 (2.8)	4.705	0.030
	Yes	212 (90.6)	104 (97.2)	–	–

*Grade 0, no pain.

#### Grade I (mild pain)

The patient experiences pain but can tolerate it, continues normal activities, and sleep remains unaffected;

#### Grade II (moderate pain)

The patient experiences significant pain that is unbearable without analgesics, and sleep is disturbed;

#### Grade III (severe pain)

The patient experiences severe, intolerable pain requiring analgesics, with severely disrupted sleep. This may be accompanied by autonomic dysfunction or positional changes.

### Analysis of compliance indicators

Variance analysi**s** of compliance indicators revealed statistically significant differences in the use of celecoxib between the two groups (*P* < 0.001). The non-day surgery group demonstrated higher compliance, with 93.2% of patients taking celecoxib regularly, compared to only 50.5% in the day surgery group. Additionally, approximately 25.2% of patients in the day surgery group did not take celecoxib at all.

There were no statistically significant differences between the groups regarding the use of Diosmin or adherence to warm water sitz baths (both *P* > 0.05). The detailed variance analysis of compliance indicators is presented in Table 4.

**TABLE 4:** Variance analysis of compliance indicators between non-day surgery group and day surgery group [*n* (%)].

Item	Value	Non-day surgery group	Day surgery group	Stats	*P*
Celecoxib	No	27 (25.2)	27 (25.2)	90.747	< 0.001
	Regularly	218 (93.2)	54 (50.5)	–	–
	As needed	1 (0.4)	26 (24.3)	–	–
Diosmin	No	15 (6.4)	10 (9.3)	1.370	0.504
	Regularly	218 (93.2)	97 (90.7)	–	–
	As needed	1 (0.4)	0 (0)	–	–
Arm water sit bath for a week	No	12 (5.1)	5 (4.7)	3.697	0.157
	Regularly, twice a day	219 (93.6)	97 (90.7)	–	–
	From time to time	3 (1.3)	5 (4.7)	–	–

### Analysis of postoperative complications

Variance analysis of postoperative complications revealed that the residual tissue prolapse rate was significantly lower in the day surgery group compared to the non-day surgery group (0.9% vs. 6.0%, *P* = 0.035).

There were no statistically significant differences between the two groups in the rates of anal stenosis, postoperative infection, anal incontinence, urinary retention (uroschesis), anal secretion, or anal itching (all *P* > 0.05).

Detailed variance analysis of postoperative complications is provided in Table 5.

**TABLE 5:** Variance analysis of postoperative complications between non-day surgery group and day surgery group [*n* (%)].

Item	Value	Non-day Surgery group	Day surgery group	Stats	*P*
Anal stenosis	No	228 (97.4)	107 (100.0)	2.793	0.095
	Yes	6 (2.6)	0 (0.0)	–	–
Postoperative infection	No	232 (99.1)	107 (100.0)	0.920	0.337
	Yes	2 (0.9)	0 (0.0)	–	–
Anal incontinence	No	228 (97.4)	107 (100.0)	2.793	0.095
	Yes	6 (2.6)	0 (0.0)	–	–
Uroschesis	No	233 (99.6)	107 (100.0)	0.459	0.498
	Yes	1 (0.4)	0 (0.0)	–	–
Residual tissue prolapses	No	220 (94.0)	106 (99.1)	4.450	0.035
	Yes	14 (6.0)	1 (0.9)	–	–
Anal secretion	No	231 (98.7)	107 (100.0)	1.384	0.229
	Yes	3 (1.3)	0 (0.0)	–	–
Itching	No	233 (99.6)	107 (100.0)	0.459	0.498
	Yes	1 (0.4)	0 (0.0)	–	–

## Discussion

Mixed hemorrhoids are a common benign anal disease that can occur at any age, with incidence increasing progressively with age. Conservative treatment primarily alleviates discomfort but is not curative, making surgery the most definitive treatment option. Among surgical methods, the procedure for prolapse and hemorrhoids (PPH) has gained rapid popularity in China due to its advantages of quicker recovery and reduced pain. Additionally, nearly all patients in the day surgery group were from the other hospitals and had recurrent disease following previous surgeries or presented with concurrent anal fissures, skin tag, external hemorrhoid and internal rectal mucosal prolapse (classified as Goligher grade III-IV). Despite complex procedure, the 30 days readmission rate in the day surgery group was 0% and the incidence of residual prolapsed tissue was significantly reduced, demonstrating that this surgical approach is safe when applied under strict selection criteria. However, PPH is not without complications, which include massive bleeding, anastomotic infections, and postoperative anal stenosis (14). Severe complications such as intestinal fistula, rectovaginal fistula, pelvic infections leading to sepsis, and even mortality have also been reported (15).

Preventing massive bleeding following PPH surgery is critical. Surgeons must refine their technical skills, ensuring that the purse-string sutures are appropriately positioned—neither too low nor too high. Sutures placed too low may rupture hemorrhoidal vessels or cause staple detachment, leading to bleeding. Conversely, excessively high sutures may limit the effectiveness of hemorrhoidal retraction. The suturing depth must reach the submucosal layer, avoiding excessively shallow or deep placement to prevent recurrence or damage to adjacent organs. Bleeding points encountered during surgery must be meticulously addressed, with secure ligatures. Routine “8”-shaped sutures across the anastomosis at 3, 7, and 11 o’clock—or even around the entire anastomosis—can also be performed as a preventive measure. Following surgery, stool softeners and anal mucosal protectants should be administered to reduce stool friction on the anastomosis and prevent bleeding.

Postoperative pain intensity was higher in the day surgery group, which may be attributed to poorer compliance with postoperative analgesia in this group. Previous studies have also reported a higher incidence of spontaneous pain following PPH compared to traditional hemorrhoidectomy (16). The non-day surgery group had a median hospital stay of 6 days, during which medication administration was strictly managed by nursing staff. As a result, 93.2% of patients in the non-day surgery group took their prescribed medication regularly. Conversely, the day surgery group with MRI-assisted diagnosis had a shorter median hospital stay of 2 days, with only 50.5% of patients taking celecoxib as directed, 25.2% not taking it at all, and the remainder using medication only as needed for pain relief.

In terms of recovery, postoperative complications, and short-term recurrence rates, this study confirmed that the day surgery model with MRI-assisted diagnosis for PPH in mixed hemorrhoids is both safe and feasible. It should be acknowledged that the diagnosis of mixed hemorrhoids is fundamentally clinical, and the role of MRI in this study was not to establish the diagnosis but to support detailed surgical planning. Through meticulous surgical techniques, we find that even complex procedures can achieve equal outcomes comparable to inpatient surgeries. Day surgery significantly reduces hospital stay duration, lowering the risk of nosocomial infections due to the shorter exposure to hospital-based pathogens. This expedited discharge allows patients to return home quickly, where they can receive more personal attention and care from their families.

Additionally, day surgery enhances hospital efficiency. It reduces surgery waiting times, accelerates ward turnover rates, and optimizes the utilization of surgical and institutional resources, thereby providing expanded medical services to the public. High-level physicians in tertiary hospitals can leverage this model to offer high-quality care, significantly shortening patient wait times and addressing imbalances in the demand and supply of hospital beds (17). It will be the first to validate the feasibility of “a dual-track day surgery model for oncology and benign diseases” in a high-volume CRC center, not to mention providing an empirical model for healthcare resource optimization.

However, day surgery also has limitations and disadvantages. It is only suitable for simple, low-risk procedures. Complex surgeries, high-risk patients, or those requiring prolonged postoperative monitoring still necessitate traditional inpatient approaches. Moreover, patients undergoing day surgery must have adequate family support to ensure proper care during recovery (18).

One disadvantage of the day surgery model is the potential for increased patient anxiety due to the absence of continuous medical monitoring. Patients may worry about their recovery and prognosis, especially given their limited hospital stay. In cases of serious complications or physical discomfort after discharge, delayed treatment could worsen the patient’s condition or even lead to irreparable consequences.

Currently, hospitals predominantly apply day surgery to small and medium-sized elective procedures with well-established techniques and manageable risks. At our hospital, the day surgery unit primarily handles conditions such as colorectal polyps, hemorrhoids, and anal fistulas. Since initiating day surgery for PPH, our surgical teams have gained valuable experience and insights through patient follow-ups.

Spinal anesthesia in day surgery carries risks including hypotension, nausea, urinary retention, and post-dural puncture headache that can delay discharge. To ensure patient safety, we implemented strict discharge criteria: stable vital signs, complete motor recovery, and spontaneous urination. These protocols prevented anesthesia-related discharge delays in all day surgery patients, demonstrating that proper safeguards are essential for successful day surgery programs.

## Key recommendations for day surgery practice

### Comprehensive preoperative assessment

A thorough evaluation of the patient’s physical condition and disease severity is essential. Attending physicians should carefully select candidates based on detailed examination results, excluding high-risk patients or those requiring extended postoperative observation. Prior to surgery, doctors should communicate clearly with patients, explaining the day surgery process, associated risks, potential complications, and all contingencies. Patients must provide informed consent and demonstrate a clear understanding of the procedure, its benefits, and its limitations. This approach minimizes conflicts and medical disputes while enhancing patient cooperation and compliance.

### Postoperative guidance and care

Patients must strictly adhere to their doctor’s instructions for postoperative care and recovery. Keeping the surgical site clean and attending periodic reviews are vital. Due to the shorter hospital stay, patients often lack access to immediate medical attention after discharge. Therefore, detailed guidance tailored to each recovery stage and possible symptoms is crucial.

To alleviate patient anxiety and facilitate a quick return to normal activities, doctors should ensure proper management of rehabilitation, pain relief, wound care, and medication use during the critical postoperative period. Establishing follow-up groups, conducting regular follow-up calls, and engaging in one-on-one conversations with patients can help address complications or discomfort promptly while improving patient compliance.

From a hospital’s perspective, adopting the day surgery model requires a high level of physician expertise and meticulous attention to patient care, ensuring both safety and efficiency.

Finally, efforts should be made to continuously enhance the quality and capabilities of medical teams, ensuring the highest level of expertise and patient safety. Additionally, strengthening the ability to handle emergencies is crucial to guaranteeing patient safety and favorable outcomes. Through continuous optimization, day surgery PPH for mixed hemorrhoids has developed unique advantages.

However, this research has several limitations. The primary limitation is the relatively small sample size. Furthermore, the time span of the study (2008–2023) may not be sufficient to comprehensively validate the findings. The earlier time frame of the non-day surgery group (2008–2023) may confound outcomes, as improvements in surgical technique, anesthesia, and hospital systems over time could have influenced results independently of the surgical model. Moving forward, we aim to expand the scope of day surgery, collect more cases, and conduct longer follow-up studies to further investigate the feasibility and outcomes of PPH for treating grade III–IV hemorrhoids. The other limitation of this study is the imbalance in surgical methods between groups, with more combined PPH and Milligan-Morgan procedures in the day surgery group (65.4% vs. 35.9%). This may confound outcomes, as combined procedures are typically more complex. Nevertheless, the day surgery group still showed favorable results, suggesting safety and feasibility even in complex cases. This may reflect selection bias or surgeon experience. Future studies should use stratified analysis or propensity score matching to reduce this confounding.

In conclusion, day surgery with MRI-assisted diagnosis for mixed hemorrhoids is feasible and associated with shorter hospitalization times, higher patient satisfaction, faster recovery, improved resource efficiency, and enhanced bed turnover.

## Data Availability

The original contributions presented in this study are included in this article/supplementary material, further inquiries can be directed to the corresponding authors.
